# CeTF: an R/Bioconductor package for transcription factor co-expression networks using regulatory impact factors (RIF) and partial correlation and information (PCIT) analysis

**DOI:** 10.1186/s12864-021-07918-2

**Published:** 2021-08-20

**Authors:** Carlos Alberto Oliveira de Biagi, Ricardo Perecin Nociti, Danielle Barbosa Brotto, Breno Osvaldo Funicheli, Patrícia de Cássia Ruy, João Paulo Bianchi Ximenez, David Livingstone Alves Figueiredo, Wilson Araújo Silva

**Affiliations:** 1 Department of Genetics at Ribeirão Preto Medical School, University of São Paulo, Ribeirão Preto, Brazil; 2 Center for Cell-Based Therapy (CEPID/FAPESP), National Institute of Science and Technology in Stem Cell and Cell Therapy (INCTC/CNPq), Regional Blood Center of Ribeirão Preto, Ribeirão Preto, Brazil; 3 Institute for Cancer Research, IPEC, Guarapuava, Brazil; 4 Laboratory of Molecular Morphophysiology and Development, Department of Veterinary Medicine, Faculty of Animal Science and Food Engineering, University of São Paulo, Pirassununga, Brazil; 5 Center for Medical Genomics, HCFMRP/USP, Ribeirão Preto, Brazil; 6 Department of Medicine, Midwest State University of Paraná-UNICENTRO, Guarapuava, Brazil; 7 Center for Integrative Systems Biology (CISBi) - NAP/USP, University of São Paulo, Ribeirão Preto, Brazil

**Keywords:** Bioinformatics, R package, R, Transcript factors, Network

## Abstract

**Background:**

Finding meaningful gene-gene interaction and the main Transcription Factors (TFs) in co-expression networks is one of the most important challenges in gene expression data mining.

**Results:**

Here, we developed the R package “CeTF” that integrates the Partial Correlation with Information Theory (PCIT) and Regulatory Impact Factors (RIF) algorithms applied to gene expression data from microarray, RNA-seq, or single-cell RNA-seq platforms. This approach allows identifying the transcription factors most likely to regulate a given network in different biological systems — for example, regulation of gene pathways in tumor stromal cells and tumor cells of the same tumor. This pipeline can be easily integrated into the high-throughput analysis. To demonstrate the CeTF package application, we analyzed gastric cancer RNA-seq data obtained from TCGA (The Cancer Genome Atlas) and found the HOXB3 gene as the second most relevant TFs with a high regulatory impact (TFs-HRi) regulating gene pathways in the cell cycle.

**Conclusion:**

This preliminary finding shows the potential of CeTF to list master regulators of gene networks. CeTF was designed as a user-friendly tool that provides many highly automated functions without requiring the user to perform many complicated processes. It is available on Bioconductor (http://bioconductor.org/packages/CeTF) and GitHub (http://github.com/cbiagii/CeTF).

**Supplementary Information:**

The online version contains supplementary material available at (10.1186/s12864-021-07918-2).

## Background

Transcriptome analysis has become crucial to identify gene circuits involved in regulating cancer hallmarks [1]. One of the intelligent ways to explore this type of data and obtain biologically relevant information about the mechanisms involved in modulating gene circuits is the inference of gene regulatory networks (GRNs). Conceptually, we can define GRN as the reconstruction of gene networks from gene expression data, revealing the connection of transcription factors (TFs) with their targets [2], aiming to highlight which gene interactions are the most relevant to the study. Despite the plethora of tools, new methods are needed to assess all possible interactions and their significance [3]. Besides, the presence of TFs in interactions for gene-to-gene is functionally crucial because they may be playing an essential regulatory role in biological processes [4]. TFs are considered key molecules that can regulate the expression of one or more genes in a biological system, thus determining how cells function and communicate with cellular environments [5]. Furthermore, integrating genome-scale and network generation with the identification of main TFs provides new insights into their data function. In this article, we provide an R package that enables performing the Regulatory Impact Factors (RIF) and Partial Correlation with Information Theory (PCIT) analysis separately, or by applying the full pipeline.

We, therefore, developed an R package called CeTF, which would not only apply the RIF and PCIT analysis, but would also perform network diffusion analysis, generate circos plots for specifics TFs/genes, functional enrichment for network conditions, and others features. The biggest advantage is that the package is intuitive to use, and the main functions are written in C/C++, which provides faster analysis for large data.

## Implementation

**Fig. 1:**
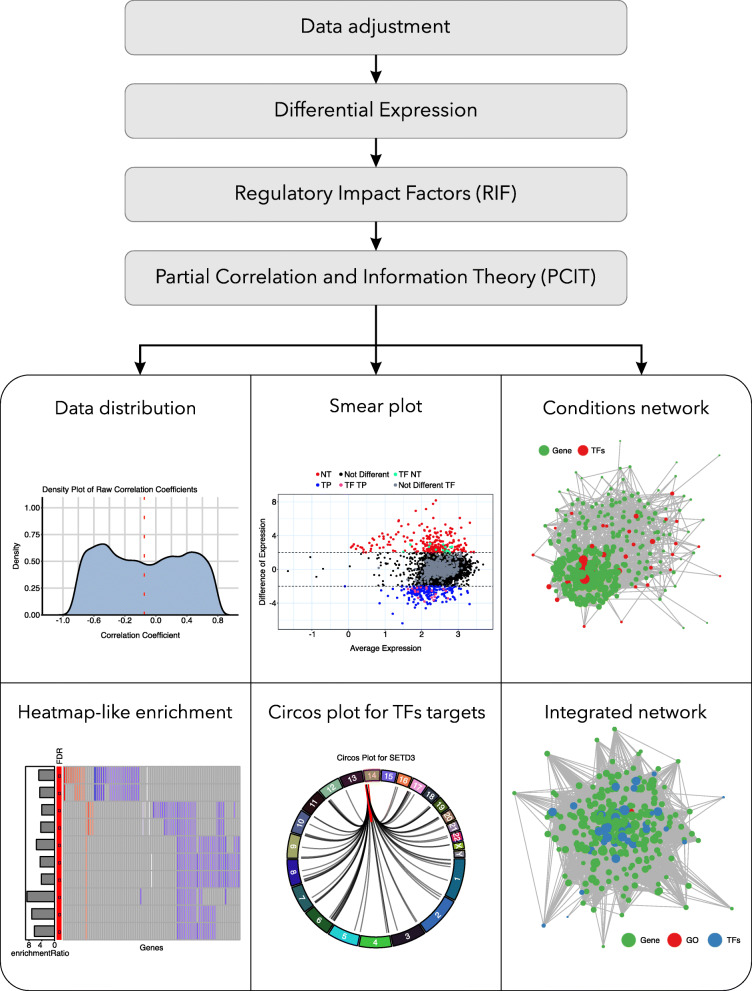
CeTF workflow. From top to bottom the four main steps start with data adjustment, followed by a differential expression, Regulatory Impact Factors (RIF) analysis and ending with Partial Correlation and Information Theory (PCIT) analysis. The plots represent visualization examples that the package can generate (i.e. data distribution, smear plot, network, heatmap, circos plot)

CeTF is an C/C++ implementation in R for PCIT [6] and RIF [7] algorithms, which initially were made in FORTRAN language. From these two algorithms, it was possible to integrate them in order to increase performance and Results. Input data may come from microarray, RNA-seq, or single-cell RNA-seq. The input data can be read counts or expressions (TPM, FPKM, normalized values, etc.). The main pipeline (Fig. 1) consists of the following steps.

### Data adjustment

If the input data is a count table, data will be converted to TPM by each column (x) as follows:

1 $$ \begin{aligned} TPM = \frac{ 10^{6}x}{sum(x)} \end{aligned}  $$

The mean for TPM values different than zero and the mean values for each gene are used as a threshold to filter the genes. Genes with values above half of the previous averages will be considered for subsequent analyses. Then, the TPM data is normalized using:

2 $$ \begin{aligned} Norm = \frac{ log(x + 1)}{log(2)} \end{aligned}  $$

If the input already has normalized expression data (TPM, FPKM, etc), the only step will be the same filter for genes that consider half of the means.

### Differential expression analysis

There are two options for differential analysis of the gene expression, the Reverter method [8] and DESeq2 [9]. In both methods, two conditions are required (i.e., control *vs.* tumor samples). In the Reverter method, the mean between samples of each condition for each gene is calculated. Then, subtraction is made between the mean of one condition concerning the other conditions. The variance of the subtraction is performed, then is calculated the difference of expression using the following formula, where s is the result of subtraction and var is the variance:

3 $$ \begin{aligned} diff = \frac{s - \frac{sum(s)}{length(s)}}{\sqrt{var}} \end{aligned}  $$

The DESeq2 method applies the Differential expression analysis based on the negative binomial distribution. Although both methods can be used on count data, it is strongly recommended to use only the Reverter method on expression input data.

### Regulatory impact factors (RIF) analysis

The RIF algorithm is well described in the original paper [7]. This step aims to identify critical Transcription Factors calculating for each condition the co-expression correlation between the TFs and the Differentially Expressed (DE) genes (from previously item). The result is RIF1 and RIF2 metrics that allow the identification of critical TFs. The RIF1 metric classifies the TFs as most differentially co-expressed with the highly abundant and highly DE genes, and the RIF2 metric classifies the TF with the most altered ability to act as predictors of the abundance of DE genes. The main TF is defined if:

4 $$ \begin{aligned} & \sqrt{RIF1^{2}} & or & & \sqrt{RIF2^{2}} & & > 1.96& \end{aligned}  $$

### Partial correlation and information theory (PCIT) analysis

The PCIT algorithm is also well described in the original paper from Reverter and Chan [6]. Moreover, it has been used for the reconstruction of Gene Co-expression Networks (GCN). The GCN combines the concept of the Partial Correlation coefficient with Information Theory to identify significant gene-to-gene associations defining edges in the reconstruction of the network. At this stage, the paired correlation of three genes is performed simultaneously, thus making the inference of co-expressed genes. This approach is more sensitive than other methods and allows the detection of functionally validated gene-gene interactions. First, is calculated for every trio of genes x, y, and z the partial correlation coefficients:

5 $$ \begin{aligned} r_{xy,z} = \frac{r_{xy} - r_{xz}r_{yz}}{ \sqrt{(1 - r^{2}_{xz})(1 - r^{2}_{yz})} } \end{aligned}  $$

And similarly, for *r*_*xz*,*y*_ and *r*_*yz*,*x*_. After that, for each trio of genes is calculated the tolerance level (*ε*) to be used as a threshold for capturing significant associations. The average ratio of partial to direct correlation is computed as follows:

6 $$ \begin{aligned} \varepsilon = \frac{1}{3} \left(\frac{r_{xy,z}}{r_{xy}} + \frac{r_{xz,y}}{r_{xz}} + \frac{r_{yz,x}}{r_{yz}} \right) \end{aligned}  $$

The association between the genes x and y is discarded if:

7 $$ \begin{aligned} & |r_{xz}| \leq |\varepsilon r_{xz}| & and & & |r_{xy}| \leq |\varepsilon r_{yz}| \end{aligned}  $$

Otherwise, the association is defined as significant, and the interaction between the genes x and y is used in the reconstruction of the GCN. The final output includes the network with gene-gene and gene-TF interactions for both conditions, besides generating the main TFs identified in the network.

### Functions of the package

There are 28 functions and 5 example datasets available in CeTF, which are described in Table 1. A working example for each of these functions is given in the package documentation in the Supplementary Material. The package allows the integration with many other packages and different types of genomics/transcriptomics analysis.

**Table 1:** Functions available in CeTF

Function	Description
bivar.awk	Summary statistics from two variables
CircosTargets	Circos plot for the Transcription Factors/genes targets
clustCoef	Calculate the clustering coefficient
clustCoefPercentage	Calculate the clustering coefficient as a percentage
densityPlot	Density distribution of correlation coefficients and significant PCIT values
diffusion	Network diffusion analysis
enrichdemo	Enrichment data
enrichPlot	Plots to visualize the enrichment analysis results
expDiff	Differential expression analysis
getData	Data accessor for a CeTF class object
getDE	Differential Expression accessor for a CeTF class object
getEnrich	Enrichment analysis for genes of network
getGroupGO	Functional Profile of a gene set at specific GO level
heatPlot	Heatmap-like functional classification
histPlot	Histogram of connectivity distribution
InputData	Input data accessor for a CeTF class object
netConditionsPlot	Network plot of gene-gene/gene-TFs interactions
netGOTFPlot	Plot a network for Ontologies, genes and TFs
NetworkData	Networks data accessor for a CeTF class object
normExp	Normalized expression transformation
OutputData	Output data accessor for a CeTF class object
PCIT	Partial Correlation and Information Theory (PCIT) analysis
pcitC	A helper to calculate PCIT implemented in C/C++
refGenes	List of reference genes for 5 different organisms to perform enrichment
RIF_input	Regulatory Impact Factors (RIF) input
RIF	Regulatory Impact Factors (RIF) analysis
RIFPlot	Relationship plots between RIF1, RIF2 and DE genes
runAnalysis	Whole analysis of RIF and PCIT
simCounts	Simulated counts data
simNorm	Simulated normalized data
SmearPlot	Smear plot for Differentially Expressed genes and TFs
TFs	Transcripition Factors data
Tolerance	Tolerance level between 3 pairwise correlations implemented in C/C++

### Additional functionalities

The CeTF package also includes additional features in order to visualize the results. After running PCIT and RIF analysis, it is possible to plot the data distribution, the distribution of differentially expressed genes/TFs that shows the average expression (in log2) by the difference of expression, the network for both conditions and the integrated network with genes, TFs and enriched pathways. Besides, it is possible to visualize the targets for specific TFs as a circos plot. It is also possible to perform the grouping of ontologies [10] without statistical inference and functional enrichment for several databases with the statistical inference of many organisms using WebGestalt database [11]. Finally, it is possible to save all tables that include interaction networks, enrichment, differential expression, main TFs, and others.

### Software construction

CeTF is an R-based toolkit, and most of the code is written in R language. PCIT and tolerance functions were written in C/C++ using Rcpp (v1.0.5) [12] and RcppArmadillo (v0.10.1.2.2) [13] for better performance. The main R packages used for analysis and visualization of the results were the circlize (v0.4.10) [14], ComplexHeatmap (v2.6.0) [15], DESeq2 (v1.30.0) [9], ggplot2 (v3.3.2) [16], RCy3 (v2.10.0) [17], and others listed in the Supplementary Material.

## Results

**Fig. 2:**
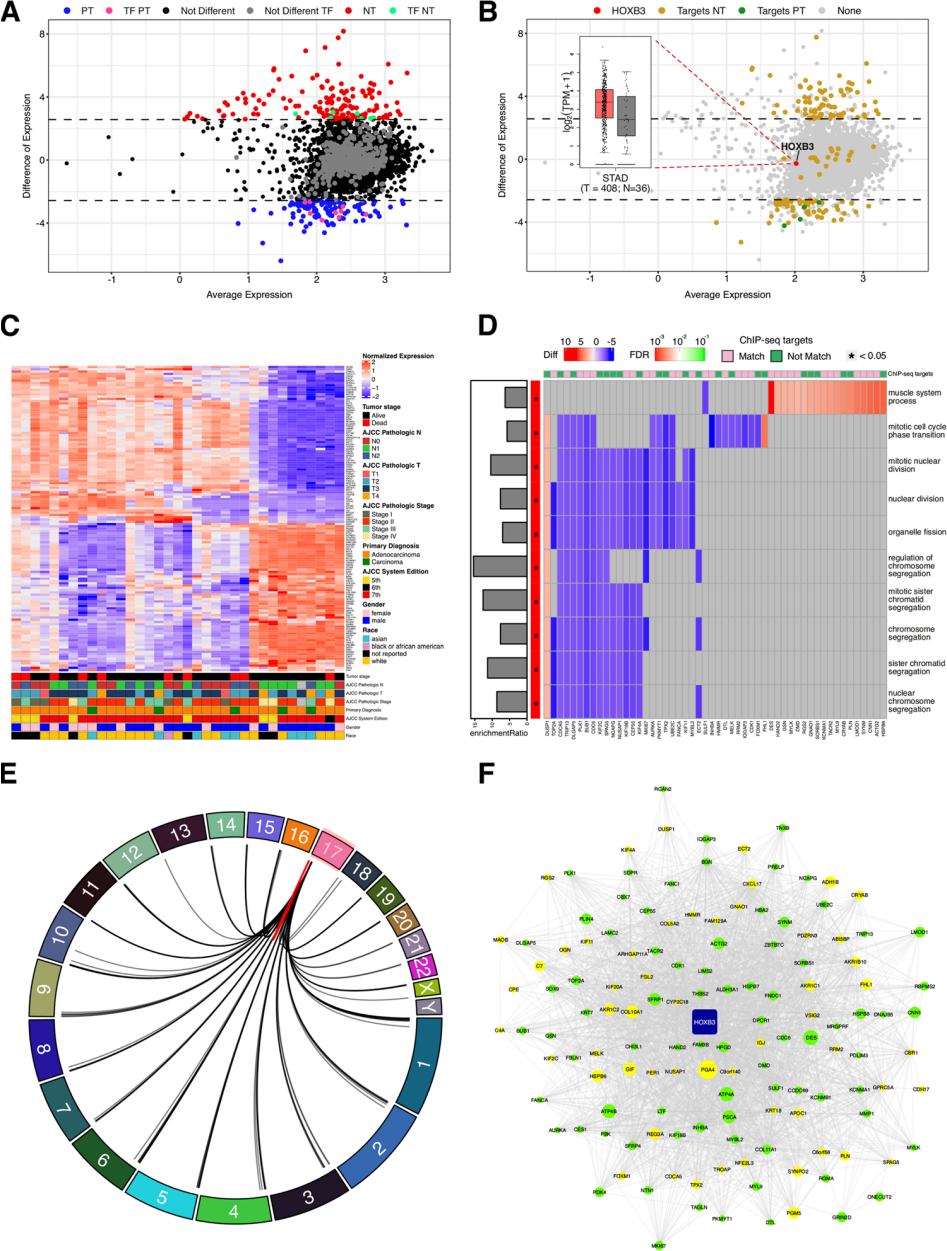
TCGA-STAD data results comparing normal versus tumor samples using CeTF. (A) Smear plot showing the difference of expression for 8,037 genes, which 151 are up-regulated (colored in red), 118 are down-regulated (blue color), and the dots in black color are not differentially expressed based in a difference of expression module cutoff of 2.57. There are 7 TFs up-regulated (green color), 9 TFs down-regulated (pink color), and 504 not differentially expressed TFs (grey color). (B) Smear plot showing the 163 HOXB3 targets. Of these, 76 are up-regulated, 58 are down-regulated, and 29 are not differentially expressed. The yellow dots represent the 149 targets associated with NT samples, and the green dots represent the 4 targets associated with TP samples. (C) Heatmap with 163 HOXB3 targets in NT samples. The bottom annotation has clinical information as tumor stage, AJCC pathologic N, AJCC pathologic T, AJCC pathologic stage, primary diagnosis, AJCC system edition, gender, and race. (D) Enrichment of 163 HOXB3 targets with Gene Ontology Biological Process showing which genes are enriched with the pathways and their expression difference. The bar plot on the left side shows the enrichment ratio. The left sidebar shows the enriched pathway significance with an asterisk if significant, a p-value less than 0.05. Finally, the top annotation shows the match between HOXB3 targets from CeTF and ChIP-seq. (E) Circos plot representing the HOXB3 targets and their chromosome position. HOXB3 is located on chromosome 17. The red line shows the 10 cis interactions (the target is located at the same chromosome HOXB3), and the black lines indicate a trans interaction (the target is locatedon a different chromosome than HOXB3). (F) Network with 134 down and up-regulated HOXB3 targets. The network has 135 nodes and 2520 edges. HOXB3 is represented in the center of the network in blue color. The green nodes represent the 79 targets found in CeTF that match with ChIP-seq targets for HOXB3 and the yellow nodes represent the 55 targets that don’t fit with them

To demonstrate the tool’s utility, we used stomach adenocarcinoma RNA-seq data from The Cancer Genome Atlas (TCGA) project [18] and applied all analyzes available in the CeTF package. Here, we compared samples from normal tissue (NT=36) and primary tumor (PT=408) of Stomach adenocarcinoma (STAD). The TFs-HRi are shown in Table 2 and the analysis of partial results in Fig. 2A.

**Table 2:** List of TFs-HRi from TCGA-STAD analysis. Here we have the Transcript Factors (*TF*) found as playing an important role in the given comparison. Also shown is the mean of expression (*avgexpr*) for each TF, in addition to the values of the metrics RIF1 and RIF2. Finally, *freq.NT* and *frep.TP* columns represent the frequency of appearance of the given TF in each condition, with *freq.diff* being the difference between these frequencies. A positive difference means that TF plays an important role in the reference condition in the NT case, whereas a negative difference means that TF plays an important role in the condition TP

TF	avgexpr	RIF1	RIF2	freq.NT	freq.TP	freq.diff
SETD3	5.854	1.409	2.189	162	13	149
HOXB3	4.309	0.517	2.282	159	14	145
RNF115	4.96	-2.324	1.64	153	19	134
TOX4	6.183	2.345	1.63	139	9	130
ASCL2	3.96	2.179	0.678	147	18	129
FOXA1	5.597	-0.801	2.022	159	34	125
SOX4	7.281	3.554	1.072	149	29	120
CSDE1	8.816	-0.069	2.153	172	53	119
TEAD3	5.903	-0.225	2.031	157	46	111
VEZF1	6.211	-0.385	2.243	157	47	110
TERF1	4.853	-2.475	0.902	123	17	106
RBBP7	7.086	2.393	1.872	147	42	105
BBX	6.22	-0.314	2.09	154	55	99
ECD	5.17	3.16	0.778	115	20	95
SPDEF	3.749	-2.081	1.078	114	20	94
TULP3	5.059	0.698	2.012	152	58	94
TRIM16	5.74	-2.266	0.721	127	35	92
ZBTB7C	3.999	-3.093	0.824	122	30	92
NFX1	5.733	3.149	0.852	96	13	83
TP53	6.305	-2.016	-0.005	89	8	81
NFE2L3	6.01	2.484	1.068	175	112	63
TSC22D4	5.989	-1.976	-0.106	72	9	63
AFF4	7.147	2.486	0.539	89	27	62
ELF1	6.758	-2.384	-0.09	76	16	60
VTN	1.905	-2.277	0.02	66	13	53
ADNP2	5.251	2.319	0.311	79	29	50
KLF4	6.519	-3.313	-0.297	73	24	49
CDC5L	5.794	2.845	-0.058	69	36	33
KLF6	7.737	-2.584	-1.222	31	10	21
PER1	5.694	2.051	0.866	127	115	12
MYC	7.064	2.127	-0.714	35	29	6
LYAR	4.775	2.242	-1.301	45	75	-30
HMGB2	6.67	-0.737	-2.214	3	66	-63
MAFB	5.29	-2.433	-1.844	32	95	-63
E2F3	4.673	0.238	-2.131	7	80	-73
SSRP1	7.323	1.431	-2.081	44	128	-84
MAF	5.527	0.495	-2.282	20	124	-104

Table 2 describes a list of 37 TFs-HRi. Among the main TFs-HRi identified, we highlight four TFs (SETD3, HOX3B, FOXA1, and SOX4) for being widely reported in association with stomach adenocarcinoma. Some studies show that high expression of the SETD3 gene is associated with poor survival in triple-negative breast cancer [19], while HOXB3 and FOXA1 were identified as indicators of better prognosis [20–22]. Interestingly, the elevated expression of the SOX4 gene has been described to regulate the epithelial-mesenchymal transition (EMT) mechanism mediated by TGF-beta [23]. The Results presented below will be centered on the HOXB3 gene, as it is one of the HOX genes studied by our group [24, 25].

After filtering data, a total of 8,037 genes remained in the analysis and are represented in Fig. 2A, with 151 up-regulated genes (red dots) and 118 down-regulated genes (blue dots). On this set of genes, 7 TFs are up-regulated (green dots), 9 TFs are down-regulated (pink dots) and 504 are not differentially expressed. Figure 2B places the HOXB3 gene as a central hub and its 2520 gene-to-gene interactions obtained with the CeTF package. Seventy-six up-regulated targets, and 58 down-regulated targets were found.

Figure 2C shows the heatmap with all 163 HOXB3 targets, which revealed no correlation with the two main groups of samples with clinical and histopathological data. A graph with the enrichment of gene pathways only with HOXB3 targets (Fig. 2D) shows that only one biological process (muscle system process) was enriched with overexpressed HOXB3 targets. Nine other biological processes were enriched with downregulated targets associated with the cell cycle, corroborating with the biology of normal tissues (Fig. 2D). Furthermore, the Chip-seq data from one of our studies (unpublished data) were used to validate the 163 targets predicted. Although the CHIP-seq data were generated from placental tissue, 54% of the targets predicted by the CeTF package have been validated (Fig. 2D). In addition to the negative control of the cell cycle, the DUSP1 gene, which is upregulated in all cell cycle biological processes, is related to the negative regulation of cellular proliferation [26]. A representation of the genomic distribution of the HOXB3 targets (located on chromosome 17) shows that the vast majority of targets are in different chromosomes. Ten targets are located on chromosome 17 (Fig. 2E). Finally, we built the network for HOXB3 and their targets (Fig. 2F). The targets validated by Chip-seq are highlighted in green color.

## Conclusions

CeTF is a tool that assists the identification of meaningful gene-gene associations and the main TFs in co-expression networks, as demonstrated previously. It offers functions for a complete and customizable workflow from count or expression data to networks and visualizations in a freely available R package. We expect that CeTF will be widely used by the genomics and transcriptomics community and scientists who work with high-throughput data to understand how main TFs are working in a co-expression network and what are the pathways involved in this context. We employ RNA-seq data of stomach adenocarcinoma from the TCGA project to demonstrate all the CeTF package analyses. We believe that the present study will help researchers either identify transcription factors with a critical role in regulating gene pathways involved with tumorigenesis or other biological systems of interest.

## Availability and requirements

**Project name:** CeTF **Project home page:**http://bioconductor.org/packages/CeTF and http://github.com/cbiagii/CeTF**Operating system:** platform independent **Programming language:** R **Other requirements:** R 4.0 or higher **License:** GPL-3 **Any restrictions to use by non-academics:** no licence needed

## Supplementary Information

**Additional file 1** Detailed tutorial for CeTF package. This file is an tutorial showing step-by-step how to use CeTF package.

## Data Availability

CeTF is a publicly available Bioconductor package available from http://bioconductor.org/packages/CeTF. Documentation is available on the Bioconductor website, and we provide vignettes describing more example analyses. We also maintain a public github repository (http://github.com/cbiagii/CeTF), and invite the community to submit or request additional functionality to incorporate into this package. This package requires R ≥4.0.0 and depends on several R/Bioconductor packages including circlize, ComplexHeatmap, clusterProfiler, DESeq2, GenomicTools, GenomicTools.fileHandler, ggnetwork, GGally, ggplot2, ggpubr, ggrepel, graphics, grid, igraph, Matrix, network, Rcpp, RCy3, S4Vectors, stats, SummarizedExperiment, utils and WebGestaltR. A web page is also available with tutorials and additional information: http://cbiagii.github.io/CeTF/. A docker image with the latest version is available in https://hub.docker.com/r/biagii/cetf.

## Abbreviations

CeTF: Coexpression for Transcription Factors
RIF: Regulatory Impact Factors
PCIT: Partial Correlation with Information Theory
TFs: Transcription Factors
TCGA: The Cancer Genome Atlas
TFs-HRi: Transcription Factors with a High Regulatory impact
GRNs: Gene Regulatory Networks
TPM: Transcripts Per Million
FPKM: Fragments Per Kilobase Million
DE: Differentially Expressed
STAD: Stomach adenocarcinoma
EMT: Epithelial-Mesenchymal Transition

## References

[1] Hanahan D, Weinberg R (2011). Hallmarks of cancer: the next generation. cell.

[2] Hu X, Hu Y, Wu F, Leung RWT, Qin J (2020). Integration of single-cell multi-omics for gene regulatory network inference. Comput Struct Biotechnol J.

[3] Yu D, Kim M, Xiao G, Hwang T (2013). Review of biological network data and its applications. Genomics Inform.

[4] Farnham P (2009). Insights from genomic profiling of transcription factors. Nat Rev Genet.

[5] Vaquerizas J, Kummerfeld S, Teichmann S, Luscombe N (2009). A census of human transcription factors: function, expression and evolution. Nat Rev Genet.

[6] Reverter A, Chan E (2008). Combining partial correlation and an information theory approach to the reversed engineering of gene co-expression networks. Bioinformatics.

[7] Reverter A, Hudson N, Nagaraj S, Pérez-Enciso M, Dalrymple B (2010). Regulatory impact factors: unraveling the transcriptional regulation of complex traits from expression data. Bioinformatics.

[8] Reverter A, Ingham A, Lehnert S, Tan S-H, Wang Y, Ratnakumar A, Dalrymple B (2006). Simultaneous identification of differential gene expression and connectivity in inflammation, adipogenesis and cancer. Bioinformatics.

[9] Love M, Huber W, Anders S (2014). Moderated estimation of fold change and dispersion for rna-seq data with deseq2. Genome Biol.

[10] Consortium G (2019). The gene ontology resource: 20 years and still going strong. Nucleic Acids Res.

[11] Liao Y, Wang J, Jaehnig E, Shi Z, Zhang B (2019). Webgestalt 2019: gene set analysis toolkit with revamped uis and apis. Nucleic Acids Res.

[12] Eddelbuettel D, François R, Allaire J, Ushey K, Kou Q, Russel N, Chambers J, Bates D (2011). Rcpp: Seamless r and c++ integration. J Stat Softw.

[13] Eddelbuettel D, Sanderson C (2014). Rcpparmadillo: Accelerating r with high-performance c++ linear algebra. Comput Stat Data Anal.

[14] Gu Z, Gu L, Eils R, Schlesner M, Brors B (2014). circlize implements and enhances circular visualization in r. Bioinformatics.

[15] Gu Z, Eils R, Schlesner M (2016). Complex heatmaps reveal patterns and correlations in multidimensional genomic data. Bioinformatics.

[16] Wickham H. Elegant graphics for data analysis (ggplot2); 2009. https://ggplot2-book.org. Accessed 18 Nov 2020.

[17] Gustavsen JA, Pai S, Isserlin R, Demchak B, Pico AR (2019). RCy3: network biology using cytoscape from within R. F1000Research.

[18] Weinstein J, Collisson E, Mills G, Shaw K, Ozenberger B, Ellrott K, Shmulevich I, Sander C, Stuart J, Network C (2013). The cancer genome atlas pan-cancer analysis project. Nat Genet.

[19] Hassan N, Rutsch N, Győrffy B, Espinoza-Sánchez N, Götte M (2020). Setd3 acts as a prognostic marker in breast cancer patients and modulates the viability and invasion of breast cancer cells. Sci Rep.

[20] Tomioka N, Morita K, Kobayashi N, Tada M, Itoh T, Saitoh S, Kondo M, Takahashi N, Kataoka A, Nakanishi K (2010). Array comparative genomic hybridization analysis revealed four genomic prognostic biomarkers for primary gastric cancers. Cancer Genet Cytogenet.

[21] Ren H, Zhang P, Tang Y, Wu M, Zhang W (2015). Forkhead box protein a1 is a prognostic predictor and promotes tumor growth of gastric cancer. OncoTargets Ther.

[22] Camolotto S, Pattabiraman S, Mosbruger T, Jones A, Belova V, Orstad G, Streiff M, Salmond L, Stubben C, Kaestner K (2018). Foxa1 and foxa2 drive gastric differentiation and suppress squamous identity in nkx2-1-negative lung cancer. Elife.

[23] Peng X, Liu G, Peng H, Chen A, Zha L, Wang Z (2018). Sox4 contributes to tgf- *β*-induced epithelial–mesenchymal transition and stem cell characteristics of gastric cancer cells. Genes Dis.

[24] Brotto D, Siena ADD, de Barros I, Carvalho SdCeS, Muys B, Goedert L, Cardoso C, Plaça J, Ramão A, Squire J (2020). Contributions of hox genes to cancer hallmarks: Enrichment pathway analysis and review. Tumor Biol.

[25] Ramão A, Pinheiro D, Alves C, Kannen V, Jungbluth A, de Araújo LF, Muys B, Fonseca A, Plaça J, Panepucci R (2016). Hox genes: potential candidates for the progression of laryngeal squamous cell carcinoma. Tumor Biol.

[26] Cheng C, Liu F, Li J, Song Q (2018). Dusp1 promotes senescence of retinoblastoma cell line so-rb5 cells by activating akt signaling pathway. Eur Rev Med Pharmacol Sci.

